# Spatially and temporally distributed data foraging decisions in disciplinary field science

**DOI:** 10.1186/s41235-021-00296-z

**Published:** 2021-04-07

**Authors:** Cristina G. Wilson, Feifei Qian, Douglas J. Jerolmack, Sonia Roberts, Jonathan Ham, Daniel Koditschek, Thomas F. Shipley

**Affiliations:** 1 Department of Psychology, Temple University, Philadelphia, PA USA; 2 Department of Electrical and Systems Engineering, University of Pennsylvania, Philadelphia, PA USA; 3 Department of Earth and Environmental Science, University of Pennsylvania, Philadelphia, PA USA; 4 Mechanical Engineering and Applied Mechanics, University of Pennsylvania, Philadelphia, PA USA

**Keywords:** Information search, Information foraging, Human–robot interaction, Metascience, Spatial heuristics

## Abstract

How do scientists generate and weight candidate queries for hypothesis testing, and how does learning from observations or experimental data impact query selection? Field sciences offer a compelling context to ask these questions because query selection and adaptation involves consideration of the spatiotemporal arrangement of data, and therefore closely parallels classic search and foraging behavior. Here we conduct a novel simulated data foraging study—and a complementary real-world case study—to determine how spatiotemporal data collection decisions are made in field sciences, and how search is adapted in response to in-situ data. Expert geoscientists evaluated a hypothesis by collecting environmental data using a mobile robot. At any point, participants were able to stop the robot and change their search strategy or make a conclusion about the hypothesis. We identified spatiotemporal reasoning heuristics, to which scientists strongly anchored, displaying limited adaptation to new data. We analyzed two key decision factors: variable-space coverage, and fitting error to the hypothesis. We found that, despite varied search strategies, the majority of scientists made a conclusion as the fitting error converged. Scientists who made premature conclusions, due to insufficient variable-space coverage or before the fitting error stabilized, were more prone to incorrect conclusions. We found that novice undergraduates used the same heuristics as expert geoscientists in a simplified version of the scenario. We believe the findings from this study could be used to improve field science training in data foraging, and aid in the development of technologies to support data collection decisions.

**Supplementary Information:**

The online version contains supplementary material available at 10.1186/s41235-021-00296-z.

## Introduction

Hypothesis testing is the engine of scientific progress. For discovering the undiscovered, there is no faster method (Platt, 1964). This is the traditional sequence of steps in experimental science, starting from a problem or situation that the scientist is attempting to understand:

1. A set of hypotheses are formed
2. A candidate set of queries (experiments, questions) are generated to assess the hypotheses
3. The scientist chooses which query to perform based on past experience
4. The query produces data which are compared to the hypotheses

In scientific practice, hypothesis testing is rarely linear. Queries can be developed before formal hypotheses, and learning from data or observations can lead to adaptation of both query selection and hypothesis formation. Understanding dynamic hypothesis testing is critical for improving scientific practice, and it can serve as a proxy for understanding how ordinary people (non-scientists) ask questions, collect information, and explore their environments (Coenen et al., 2019).

In this paper, we address two specific aspects of hypothesis testing: how scientists generate and weight candidate queries, and how learning from observations or experimental data impacts query selection. We focus on hypothesis testing in field sciences, using a sample of expert geoscientists. Field sciences, and geoscience especially, offer a compelling context to assess hypothesis testing because query selection and adaptation involves consideration of the spatiotemporal arrangement of data—i.e., what areas have the highest data value, and what are the costs required to find and extract data? This spatiotemporal element of hypothesis testing closely parallels classic search and foraging behavior, allowing us to draw from a rich body of work on foraging to inform our understanding of scientific query selection and adaptation in response to new information (e.g., Fagan et al., 2013; Pagliara et al., 2018; Viswanathan et al., 2011). As field scientists are increasingly embracing technologies that provide in-situ data, such as mobile robots (Qian et al., 2017, 2019; Wei et al., 2020), it is critical to determine how the availability of new information influences adaptive data collection decisions.

The paper is organized as follows. First, we briefly review the foraging literature on location selection and adaptation, and use this information to form predictions about how geoscientists might select and adapt data collection strategies, or *data forage*. Next, we describe a field case study of how one expert geoscientist selected and adapted data collection strategies using in-situ data from a mobile robot. Then, we present findings from our research using a simulated geologic decision scenario, in which participants must evaluate a given hypothesis by selecting an initial data sampling strategy and then adjusting that strategy in response to incoming data. We compare the strategies of expert geoscientist participants with novice undergraduate participants, drawing general conclusions about query selection and adaptation in science and its implications for both scientists and ordinary populations.

This work anticipates a near future where humans and robots operate in coordination to explore, collect scientific data and test hypotheses. If we want to build and deploy embodied intelligent agents that are capable of making successful science-driven decisions, then it is fair to say that models of how humans make such decisions are a good place to start. The research and case study presented in this paper represent our interdisciplinary teams’ first attempts at characterizing expert field scientist choice behavior. The combination of traditional “laboratory” research with naturalistic observation is unusual, but this approach is intentional—understanding human decision making in the wild will require both the rigor and precision of lab-based techniques, and the open-ended potential of observational, naturalistic study.

## Background and hypotheses

When foraging, it is unmanageable for human decision makers to generate and weigh *all* future outcomes or scenarios during initial location selection; instead, they must approximate the search space in a timely and computationally efficient manner. In a familiar environment, where the value of different resources and their respective probability of occurring at a site are known, this can be accomplished via simple rate maximization^1^ (Pyke, 1984). Yet, often site selection in foraging and field science is characterized by a high degree of uncertainty: the search space may be large, and the probability of site value ambiguous or unknown, making it computationally difficult or impossible to weigh candidate sites for comparison and, ultimately, selection.

Human foraging under uncertainty has been studied via laboratory tests in simulated environments (often computer based: Ehinger & Wolfe, 2016; Hills et al., 2013; Wilke et al., 2015, but cf. Maya et al., 2019), and through naturalistic observation of hunter-gatherer populations (Berbesque et al., 2016; Pacheco-Cobos et al., 2019). The majority of this work has dealt with foraging from patchy distributions—resources (signals) that occur in clumps in the environment (noise). Patchiness occurs more frequently in natural environments than randomness or dispersion (Taylor et al., 1978), and as a result human and non-human animals have adapted successful rules-of-thumb for continuing to exploit resource patches while they are still profitable (Charnov, 1976; Hutchinson et al., 2008; Wilke et al., 2015).

In contrast to traditional foraging, scientific data collection (or *data foraging*) involves detection of an underlying pattern in the environment system (e.g. a signal gradient). A key insight is that, within an environment system, the underlying pattern is not necessarily uniformly distributed and data can be of varying value. Thus, field scientists’ data foraging decisions aim to capture complex patterns in the natural system, where the distribution of information value is uncertain—this can be conceived of as detecting not just the presence of patches, but also the meaningful organization across patches in uncertain space. Successful data foraging strategies characterize the system without “cherry-picking” data and thus biasing interpretation (e.g., focusing only on a subset of the observed data, such as observations that are consistent with a predicted pattern). The purpose of this paper is to ask how expert scientists make data foraging decisions when searching for a hypothesized signal gradient, where the expectation is that “resources” (data) will be distributed non-uniformly over the gradient and the goal is to collect data in an unbiased manner.

Under conditions of uncertainty, the foraging literature and the literature on judgment and decision making both suggest that humans will rely on learned rules-of-thumb (heuristics). Humans and other animals possess sophisticated cognitive capabilities that allow them to learn from previous experience and form mental shortcuts to navigate their environments in a non-random way^2^ to optimize gains (Fagan et al., 2013; Moser et al., 2008). Experience and socialization impact the acquisition of foraging skills (Maya et al., 2019; McElreath & Koster, 2014; McElreath et al., 2008), and in a similar fashion, field scientists can learn heuristic strategies for data collection through experience with field work or via social transmission, from mentor to mentee or among members of a sub-discipline.

In the field case study and research presented below, we sought to characterize the data foraging heuristics that expert scientists rely on when faced with uncertainty about underlying spatial gradients in the environment. As field researchers tend to agree with each other on the appropriate methods for sampling data (e.g., Reverdy et al., 2017) we anticipated that experts’ heuristics would not be idiosyncratic; that is, experts would likely employ a constrained set of heuristics. Environmental and geological sampling authorities recommend uniform sampling to avoid location bias (U.S. Environmental Protection Agency, 2002; Wolman, 1954), as well as taking an equal number of measurements at each location to handle measurement error and natural variability (Geboy & Engle, 2011; U.S. Geological Survey, 1987). Our aim was to determine (1) if practicing scientists actually follow these heuristics, (2) whether the heuristics make sense for the structure of environmental gradients, and (3) whether the heuristics are quantitatively better or worse at capturing high-value data than random strategies or strategies used by novices. This has important implications for scientific training in data search—i.e., does scientific training promote data collection heuristics that are efficient and effective?—as well as the use of robots for autonomous data collection—i.e., is science best served by robots that collect data according to the principles of the human expert mind, or some other method, like random sampling? By using a novice sample (no geology experience), we can also begin to address the question of whether the data foraging heuristics scientists apply are learned and domain-specific, or are representative of a more general approach to spatiotemporal search problems.

We were also interested in exploring if and how experts updated their heuristics in response to new incoming data. From the foraging literature, one possibility is that encounters with valuable resources in the environment will trigger adaptation of search strategy and local intensive search behavior (referred to as encounter-conditional search or area-restricted search; Hills et al., 2013; Pacheco-Cobos et al., 2019). In the simulated geologic decision scenario used in the current study, area-restricted search would present as abandoning initial foraging heuristics in response to measurement data from a new location, followed by intensive sampling at or around this “triggering” location. An alternative possibility is that experts rely too heavily (or “anchor”) on initial foraging heuristics and fail to appropriately adapt foraging in response to incoming data. The cognitive literature on judgment and decision making shows that anchoring is a robust and pervasive effect in human decisions, both amongst novices (Furnham & Boo, 2011; Tversky & Kahneman, 1974), and discipline experts (Brewer et al., 2007; Enough & Mussweiler, 2001). Thus, in the field case study and research presented below, we consider both experts’ initial data collection strategies and how they alter strategies as new data become available that are relevant to a hypothesis.

## Field case study

**Fig. 1:**
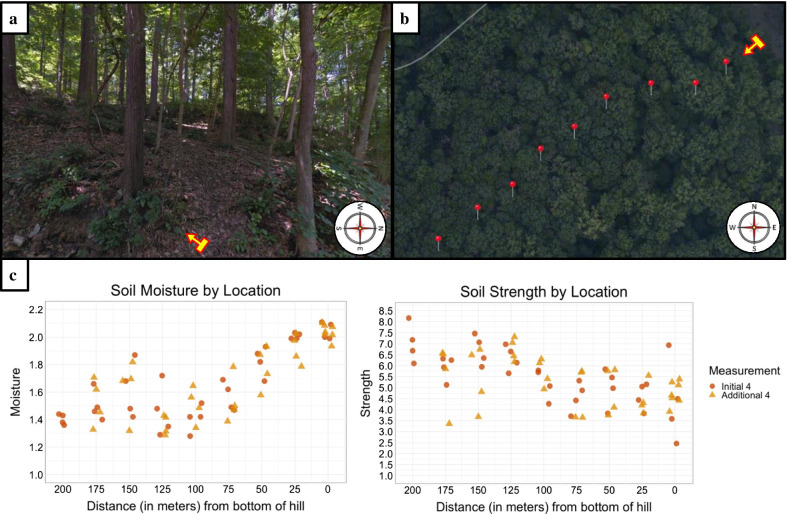
**a** Field site in Wissahickon Valley Park where expert geoscientist case study participant received surface-soil intrusion strength data and soil moisture data in situ. The yellow arrow, located at the bottom of the hill, points uphill to the SW along the hill transect where data was collected. **b** Aerial view of the hill transect, with the yellow arrow in the same position and pointing in the same direction as in A. The expert geoscientist had an initial strategy of taking four measurements each at nine evenly spaced locations. **c** After the expert saw the variability in the first four measurements (represented by red circles) at each location in situ, he opted to adjust his initial strategy and take four additional measurements (represented by yellow triangles)

The field case study was run with one geoscientist participant at a location in the Wissahickon Valley Park, a forested hillslope environment in Philadelphia, Pennsylvania (see Fig. 1a). The geoscience goal was to assess how soil strength changed over a spatial gradient and the expert participant was assisted in hypothesis testing by the data-collection robot RHex.^3^ RHex, its capabilities and geoscience applications, are discussed in greater detail in Sect. 4. The particular case study site within the Wissahickon Valley Park woods was selected because it was accessible, contained a transect from valley to ridge that could be traversed by both robot and human, and was broadly representative of the hillslopes in the region (elevation monotonically increasing from valley to ridge).

Hypotheses about soil strength in a forested environment are complex because forest soil is heterogeneous in chemical composition and particle size, with three distinct components: sand resulting from the breakdown of local rock, clay that accumulates gradually (over millennia) from chemical weathering of rock, and organic matter associated with vegetation. Moving from a ridge top to a river-valley bottom, the geoscientist generally expected soil would hold increasing moisture, and a gradual enrichment of clay and organic matter would make soil less strong (as measured via robotic leg intrusion), i.e., the moist porous aggregates at the bottom of the hill would compact under pressure making intrusion easier, while the dry sand at the crest would be hard to penetrate. However, the geoscientist noted that local variations in bedrock exposure, tree-root density and drainage could be large enough to overwhelm the expected trend. Also, because soil moisture covaries with grain size (finer particles are more poorly drained), it may not be possible to isolate these two potential controlling variables.

To evaluate his hypothesis about soil strength over the hillslope, the geoscientist selected nine evenly spaced locations (approx. 40 m apart) with four soil strength, soil moisture, and grain size measurements taken at each location (see Fig. 1b). Soil strength measurements were collected and reported in-situ by the robot RHex, driven by a human operator, while soil moisture measurements and soil samples were collected by a human field assistant. Soil samples were used to determine grain size later in the laboratory, so only soil moisture and soil strength information was available in-situ. The geoscientist was presented with the data one location at a time. At each location, after seeing the initial four measurements for moisture and soil strength plotted by distance on the transect, the geoscientist opted to take an additional four measurements of each. This continued until location eight, at which point inclement weather forced an early conclusion of the field day.

The reason the geoscientist took additional measurements was because he was unsatisfied that the initial four were sufficient to capture variability at a particular location (seeFig. 1c). Ultimately, denser sampling at each location revealed a clear moisture trend that supported the geoscientist’s expectations, i.e., steady decline in moisture moving from the bottom of the hill towards the crest. Denser sampling also revealed large variability in soil strength measurements within location—locations towards the bottom of the hill produced noisy low average soil strength measurements, and locations towards the top produced less noisy and relatively higher average strength measurements. This pattern of soil strength results provides general support for the hypothesis that soil strength would increase from the bottom of the hill to the crest. However, the geoscientist indicated he would want to collect additional data on the same transect before reaching a conclusion about the hypothesis—focusing data collection on the transition zone of the hill slope, where soil strength appears to make the jump from low to high, and moving perpendicular to the transect to get a high density of measurements in this area of rapid change. These data collection strategies were executed in subsequent field outings with RHex to the same location, ultimately resulting in a high spatiotemporal resolution dataset of soil dynamics that served as “ground-truth” for this particular hillslope.^4^ The dataset revealed that the data collection strategy initially used by the geoscientist in the case study, choosing evenly spaced locations and taking a consistent number of samples at each location, produced data that were representative of the overall pattern of soil strength and soil moisture across the hillslope (as defined by ground-truth).

## Simulated geologic decision scenario

**Fig. 2:**
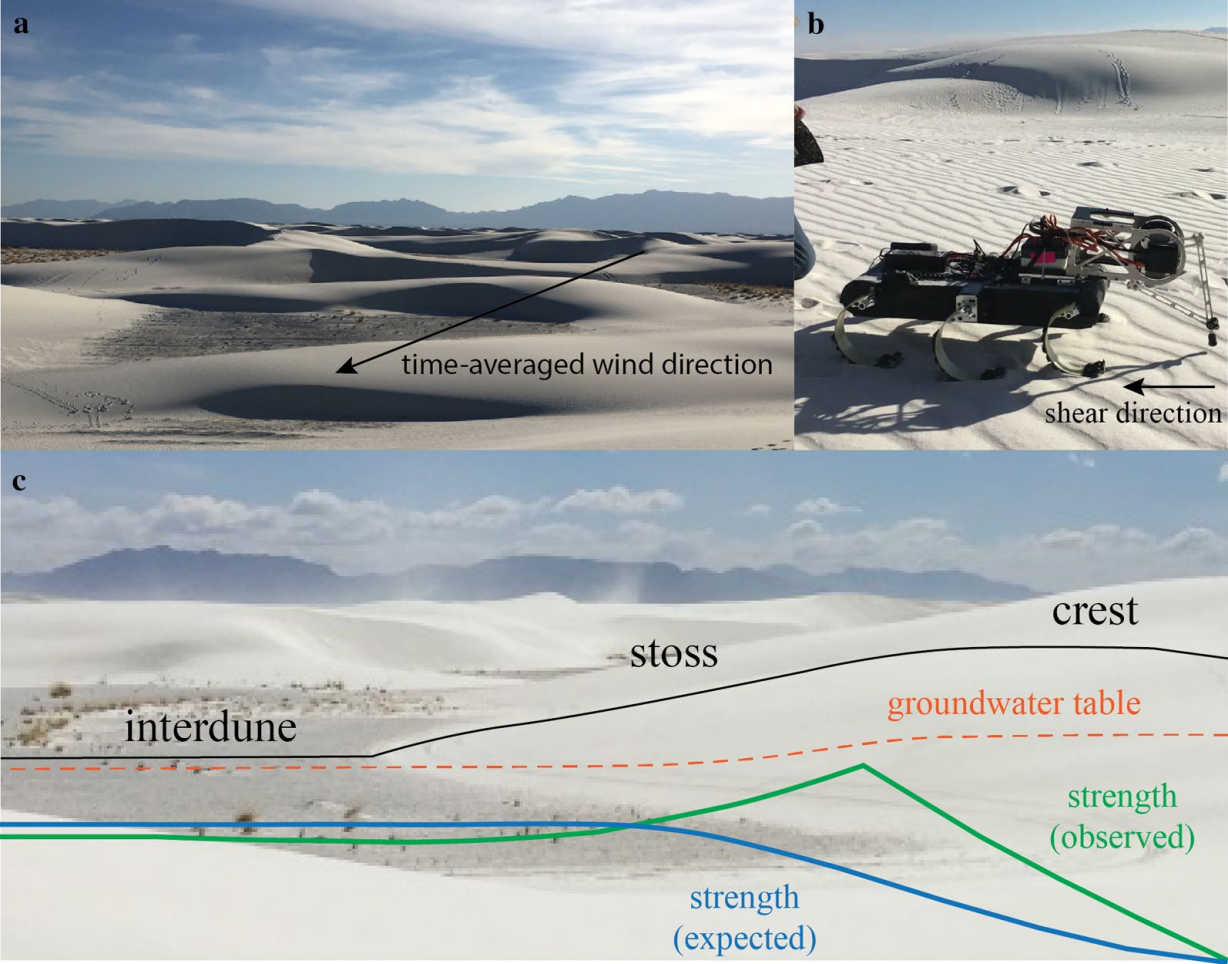
**a** Sample field site in White Sands, NM where geoscientists used the robot RHex (Qian et al., 2017) to measure surface-soil shear strength (inversely related to and hence a proxy for erodibility) along dunes with sharp moisture gradients. **b** For each shear test, the leg penetrated a few millimeters into the sand, and then dragged a thin layer of grains across the surface while measuring the mechanical shear strength of the sand. See Additional file 1 for a video of RHex performing the shear test. **c** Transect (black line) of a dune where measurement data were collected (picture from Qian et al., 2019). At the crest of the dune, where soil was driest because of its distance from the groundwater table (orange line), shear strength was expected to be low. As moisture increased on the stoss face moving towards the interdune, shear strength was expected to also increase before leveling off at the point of moisture saturation. This pattern of expected results is displayed in blue. Instead, geoscientists observed that shear strength decreased slightly as soil became more saturated nearing the interdune area just before levelling off (green line)

Inspired by the case study, we created a simulated geologic data collection scenario, where the aim was to test the generality of the query selection and adaption strategies used by the expert geoscientist in the field. We opted to stage our simulated scenario in a different environment—White Sands National Monument, New Mexico (see Fig. 2a)—where previous field work with the semi-autonomous hexapedal robot RHex revealed an unexpected discovery of a more complex relationship between soil moisture and soil strength, made possible by the ability to adjust data collection in situ.

The aim of the field trip to White Sands, led by authors Jerolmack and Qian, was to use RHex to examine wind-blown sand transport, the dominant geologic process in this landscape (Qian et al., 2017). In this field site there are sharp spatial gradients in soil moisture and plant density across dunes. Moisture and plants change a dune’s susceptibility to wind erosion, termed *erodibility*, by binding sand grains together. Previous research by McKenna-Neuman and Nickling (1989) found that the threshold wind speed for sand erosion increased with increasing soil moisture from 0 to 1%, and that this effect leveled off at approximately 2% soil moisture.

Dunes at White Sands present a natural soil moisture gradient (see Fig. 2C); a shallow groundwater table (orange dotted line) means that low-elevation interdune areas have relatively high soil moisture, and this moisture decreases as one traverses up a dune to the top crest (Jerolmack et al., 2012). The robot, RHex, performed a "plowing" test of surface-soil shear strength (see Fig. 2B). For each test, the robot leg penetrated a few millimeters into the sand, and then dragged a thin layer of grains across the surface while measuring the mechanical shear strength of the sand (see Additional file 1 for a video of RHex performing the shear test). The data from RHex showed shear strength was lowest on the dune crest where the soil was driest, as expected. Shear strength increased along the stoss towards the interdune as soil moisture increased from 0 to 3%, also as expected. Beyond 3% soil moisture, however, shear strength decreased slightly as soil became more saturated nearing the interdune area. This last result was not expected based on previous research (McKenna-Neuman & Nickling, 1989)—on Fig. 2C, the expected (blue) versus observed (green) erodibility gradient is shown. Importantly, the discovery of a more complex relationship between soil moisture and soil strength was only made possible by the ability to make multiple measurements of the strength of complex soils in situ, using a custom instrument with much more measurement sensitivity than previous approaches (Qian et al., 2019).

### Procedure

In the decision scenario, participants were asked to imagine they were studying the relationship between sediment moisture and shear resistance at White Sands. The shear resistance "plowing" test executed by RHex was described. The provided hypothesis was that moisture and shear resistance increase until sand is saturated, at which point shear resistance is constant as moisture increases; i.e., the McKenna-Neuman and Nickling (1989) hypothesis described above and shown in Fig. 2c in blue. We gave participants this hypothesis because it represents a common-sense view of the relationship between moisture and sediment strength.^5^ Because the goal of our research was to determine how scientists select and adapt data collection strategies during hypothesis testing, it was important that all participants start with the same initial hypothesis; individual differences in hypothesis generation could have a downstream effect on query generation, selection, and adaption. Participants were instructed to collect data only to evaluate the provided hypothesis, and were not asked to generate or test alternative hypotheses.

**Fig. 3:**
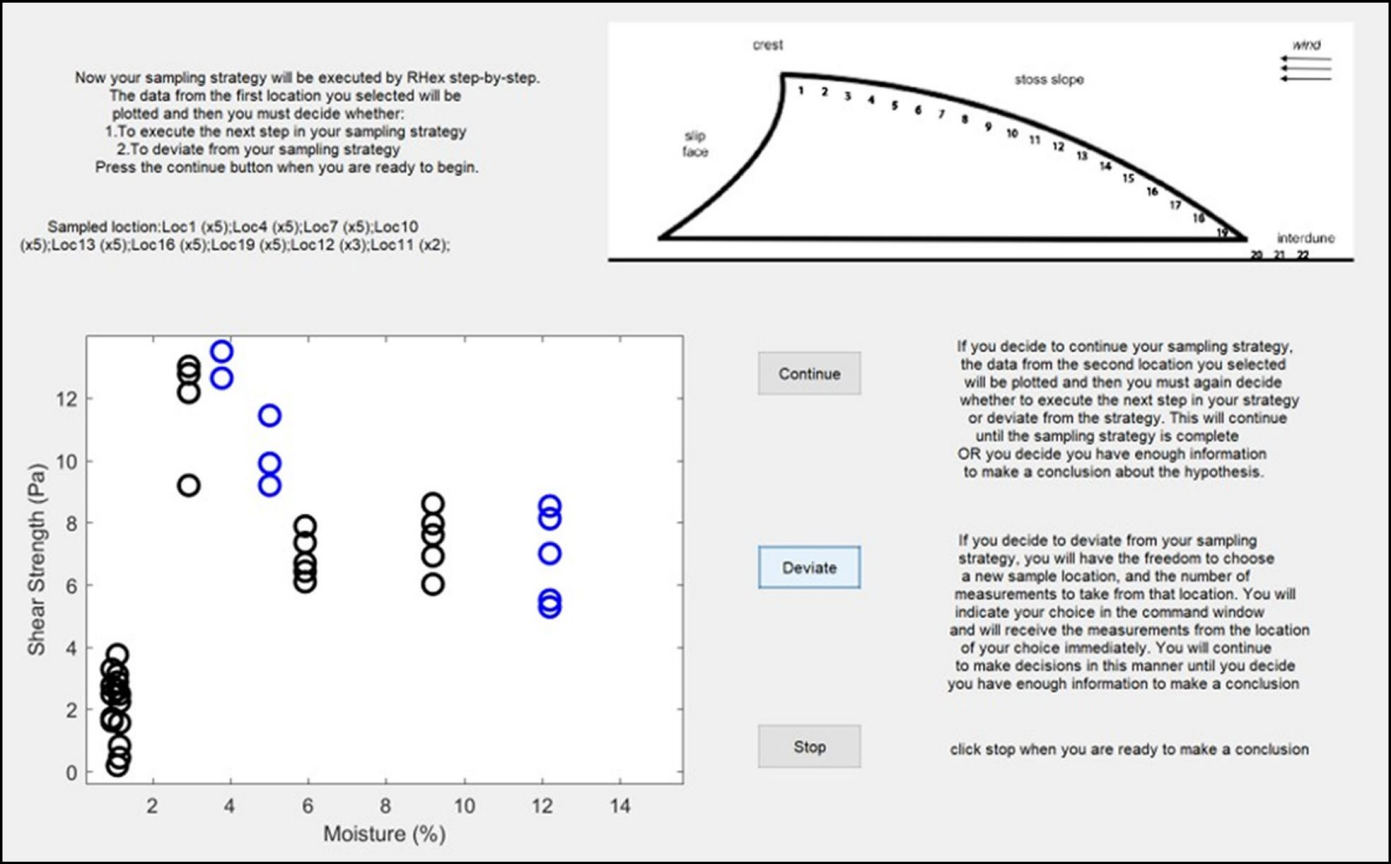
Decision-making scenario MATLAB user interface. After reporting an initial sampling strategy to the experimenter using the diagram of the dune cross-Sect. (22 possible locations, up to 10 measurements at each location), participants plotted their measurement data one location at a time by pressing the “Continue” button. Data points appeared in black on the moisture-strength scatterplot (lower left), and visited locations were recorded in text just above the scatterplot along with the number of measurements taken, e.g., Loc1 (× 5). Participants could quit their initial strategy at any point by pressing the “Deviate” button and reporting a new location to the experimenter—data from this location were plotted immediately (in blue). When participants were ready to make a conclusion about the hypothesis they pressed the “Stop” button. The user interface code is available online: https://osf.io/yhpxs/

Participants evaluated the hypothesis by collecting data along a single dune transect. The scenario user interface is shown in Fig. 3. First, participants were asked to report an initial sampling strategy by (a) identifying all sample locations on the diagram of a dune cross-section, and (b) indicating the number of measurements they wished to take at each location. There were 22 locations to choose from, with up to 10 measurements at each location. Participants were allowed to sample in any spatial order, but were instructed to be efficient and select a strategy that reflected how they would behave in similar situations in the real-world. Participants’ sampling strategy was then “executed” by RHex, with the raw measurement data for each location being plotted on screen for the participant one location at a time, in the selected order. At any point, participants were allowed to quit their initial sampling strategy and make a conclusion about the hypothesis, or change their initial strategy and collect additional data (either at a new or previously visited location). Participants were also allowed to collect additional data at the conclusion of their initial sampling strategy.

**Fig. 4:**
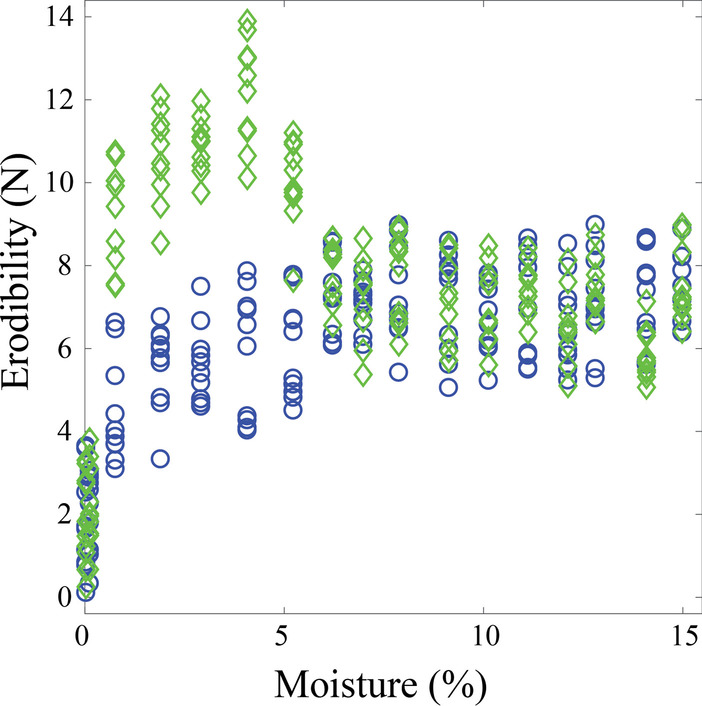
Datasets corresponding to the given hypothesis (blue circles) and the alternative-unknown hypothesis (green diamonds). For each dataset, 220 measurements are plotted reflecting the maximum of 10 measurements at 22 locations. Participants only saw a subset of the complete dataset—data were randomly selected and presented from the larger distribution based on each participants’ strategy. See Additional file 1 for a more complete description of how each dataset was computationally generated

Unbeknownst to participants, they were randomly assigned to either receive data from an underlying distribution that supported the McKenna-Neuman and Nickling (1989) hypothesis, or receive data from a distribution that mimicked the results of the Qian et al. (2019) study (see Fig. 2c, blue and green patterns of erodibility, respectively). For each hypothesis, we created a dataset of soil strength versus moisture content for 22 locations along the transect, with 10 measurements available per location. We generated the 10 measurements at each location with a truncated normal distribution around the average soil erodibility and moisture. For erodibility, we used a standard deviation of ± 2 with truncating a bound of ± 2, whereas for moisture we used a standard deviation of ± 0.5 with a truncating bound of ± 1. The gaussian distributions were truncated to reduce variability in the experiences among observers by removing chance encounters with low probability outliers. See Additional file 1 for a more complete description of how each dataset was computationally generated. The full datasets are shown in Fig. 4.

The participants were asked to make a judgment to either accept or reject the given hypothesis once they felt they had enough data to make a conclusion. Participants provided a rating of judgment confidence (the options were: very confident in my conclusion, moderately confident, slightly confident, not at all confident) and were asked to report their familiarity with the hypotheses or field setting, and what future steps, if any, they would take to improve confidence in their conclusion. Participants also completed a brief demographic questionnaire.

### Participants

We recruited 41 expert geoscientists (11 women) and 84 novice undergraduates (75 women) as participants for this study. Participants had to be at least 18 years of age. Expert geoscientists were recruited at the 2018 Fall meeting of the American Geophysical Union or through personal contact with the authors. An expert status in geoscience was defined as completion of a bachelor degree in a geoscience-related field; two participants were removed prior to analysis because they had not yet completed their bachelor degree. Geoscientist participants completed the decision scenario exactly as outlined in the preceding section. Twenty experts (4 women) were randomly assigned to sample data from a distribution supporting the given hypothesis (i.e., McKenna-Neuman & Nickling, 1989), and the other 19 experts (8 women) sampled from a distribution supporting the Qian et al. (2019) finding (see Table 1). Experts ranged in age (Min = 22, Max = 81, Med = 34, SD = 12.47), and in their years of experience post-bachelors (Min = 0.5, Max = 60, Med = 10, SD = 12.3).

**Table 1:** Random assignment of geoscience expert and novice undergraduate participants to task conditions

	**N**
*Geoscience experts*	39
Sampled from data supporting given hypothesis	20
Sampled from data supporting alternative-unknown hypothesis	19
*Novice undergraduates*	84
Limits on both location selection and number of measurements	29
Limits on location selection, but not number of measurements	29
No limits on location selection, nor number of measurements	26

Novice undergraduates were recruited through the Temple University Psychology Subject Pool. Most undergraduates were between the ages of 18 and 21 (Min = 18, Max = 49, Med = 19, SD = 3.59) and were social science or health science majors (see Table 1). Undergraduate participants completed a simplified version of the decision scenario, where they were given the same hypothesis and only asked to form an initial data collection strategy. Undergraduates were provided with the exact same image of the dune cross-section and instructed to use the image to select a sampling strategy to evaluate the given hypothesis. No measurement data were provided and participants were not given the opportunity to revise their strategy. Approximately one third of undergraduates (*n* = 29) completed a version of the scenario where they were limited to taking measurements at specific labeled locations and limited to 10 measurements at each location (akin to initial strategy selection constraints placed on expert geoscientists). One third (*n* = 29) completed a version of the scenario with no limitations on the number of measurements, and the final third (*n* = 26) completed a version with no limitations on measurements or locations. We wanted to know whether undergraduates who completed a scenario with the same constraints as the geoscience experts (labeled locations, maximum measurements) would choose similar strategies to undergraduates who completed a scenario with no constraints on location or measurement. This allows us to better judge the extent to which constraints on measurement and location may have influenced expert decisions.

### Results

First, we examined whether geoscientists’ initial data collection strategies were heuristic-driven. As expected, we found strong evidence of heuristic strategies in the selection of location and number of measurements. Approximately 87% of geoscientists (34 of 39) selected locations with roughly uniform intervals, and*all* geoscientists selected a constant number of measurements to take at each location. From here on out, we refer to these as the *equal spacing heuristic* and the *magic number heuristic*,^6^ respectively.

**Fig. 5:**
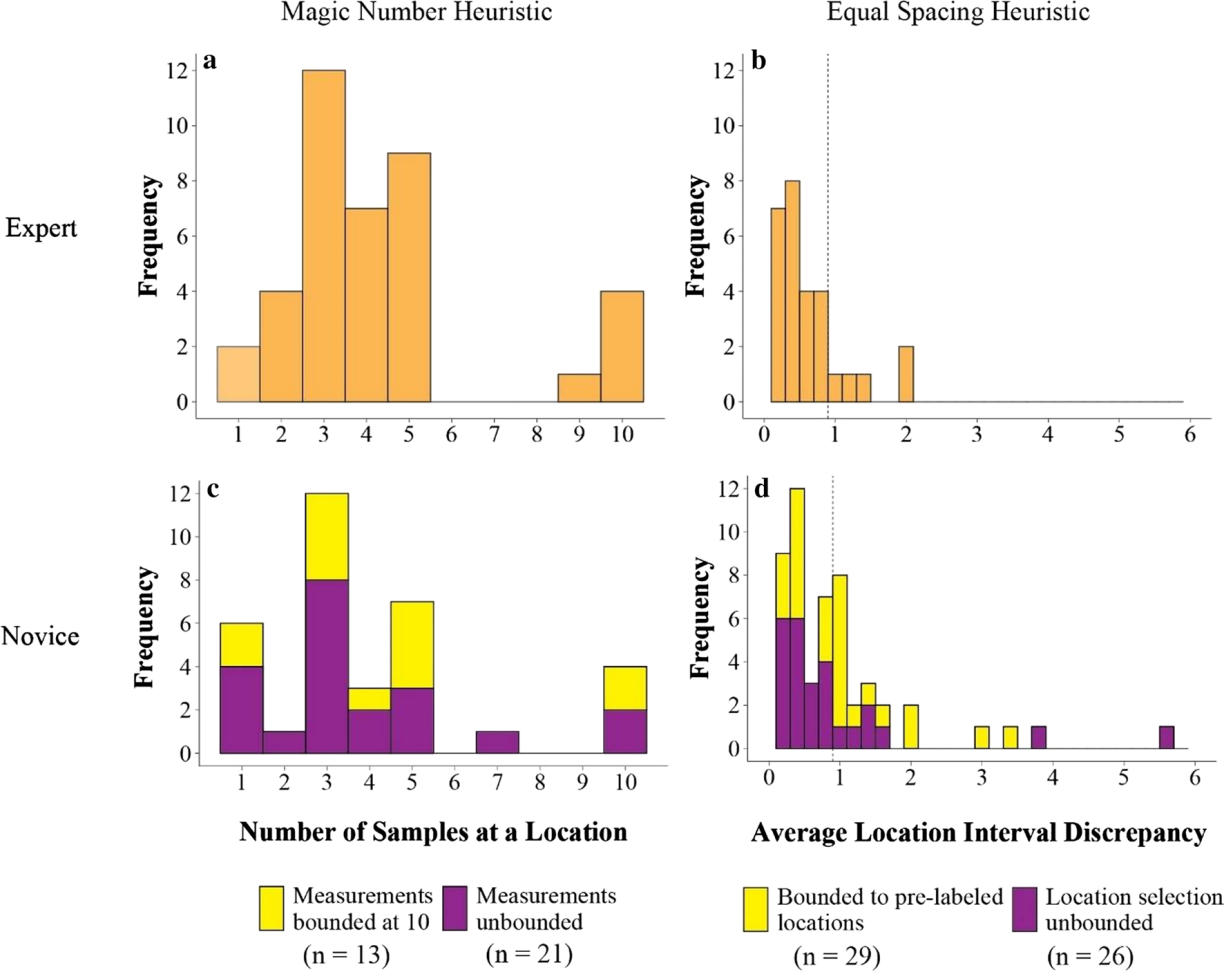
Number of measurements taken at each location during initial strategy selection (magic number heuristic, right column) and average location interval discrepancy (equal spacing heuristic, left column) in expert geoscientist (top row) and novice undergraduate participants (bottom row). Average location interval discrepancy was computed by ordering interval sizes smallest to largest and taking the mean difference of intervals—a score of zero indicates perfectly uniform location intervals, and a threshold of one (dotted vertical line in plots B and D) was used to identify participants who selected non-uniform intervals. Novice undergraduates were randomly assigned to complete either bounded or unbounded versions of the task—participants were limited to 10 measurements per location and selecting from pre-labeled locations, or the number of measurements and location selection was unconstrained. Bounded versus unbounded conditions were included to test the extent to which constraints on measurement and location may have impacted sampling strategy

The expert distribution of magic numbers is shown in Fig. 5a. Most geoscientists selected a magic number between three and five.^7^ The expert distribution of spacing strategies, characterized by the average interval discrepancy, is shown in Fig. 5b. An average interval discrepancy of zero indicates perfectly uniform location intervals, and a threshold of one was used to identify participants who selected non-uniform intervals. Amongst those who went against the equal spacing heuristic and opted for non-uniform intervals, there was no consistent background, experience level, or specialization.

Novice undergraduates also showed reliance on the equal spacing and magic number heuristics during initial strategy selection, but to a lesser degree than expert geoscientists. Approximately 63% of undergraduates (53 of 84) selected locations at roughly uniform intervals, and 60% (51 of 84) maintained a consistent number of measurements at each location. Among those undergraduates who relied on a magic number, the distribution was roughly similar to expert geoscientists, with most novices selecting a magic number between three and five, as shown in Fig. 5c. This suggests that the magic number and equal spacing heuristics (adhered to by almost all experts, but only a subset of novices) might be behavioral tendencies that are reinforced in field science practice, through either experience or cultural transmission.

Note also in Fig. 5c and d that the distributions of magic numbers and equal spacing are similar between novice undergraduates in the bounded versus unbounded conditions. Undergraduates who were told they could take as many measurements as they wished (unbounded measurement) ended up taking a similar range of measurements as those who were told they could take a maximum of 10 per location (bounded measurement). Undergraduates who were allowed free choice of locations along the transect (unbounded location) ended up equally spacing their locations in a similar fashion to those who were instructed to take measurements at pre-labeled locations (bounded location). From these results we infer that constraints on labeled locations and maximum measurements in the expert scenario likely had minimal impact on the expert distributions of magic numbers and equal spacing.

Next, we were interested in determining whether the magic number and equal spacing heuristics employed by expert geoscientists and (some) novice undergraduates led to increased likelihood of collecting high value data relative to random sampling strategies. To accomplish this, we ran 100 iterations of 2 popular heuristic strategies (3 measurements at 8 evenly spaced locations, 3 measurements at 11 evenly spaced locations), and 2 corresponding random strategies (3 measurements at 8 randomly selected locations, 3 measurements at 11 randomly selected locations). Iterations were run on both datasets (corresponding to the given hypothesis and the alternative-unknown hypothesis), and for each of the resulting generated datasets the hypothesis fitting error was determined. To compute the fitting error, the given relationship between erodibility $\left( y \right)$ and moisture $\left( x \right)$ was represented as a piecewise linear function:

$$y = \{ kx,x < b a,x \ge b$$

where $a$ represents the stabilized erodibility, $b$ represents the saturation moisture content, and $k$ represents the slope of erodibility versus moisture before saturation. The fitting error between the data and the given hypothesis was computed as the root mean squared error (RMSE) of this linear regression.

**Fig. 6:**
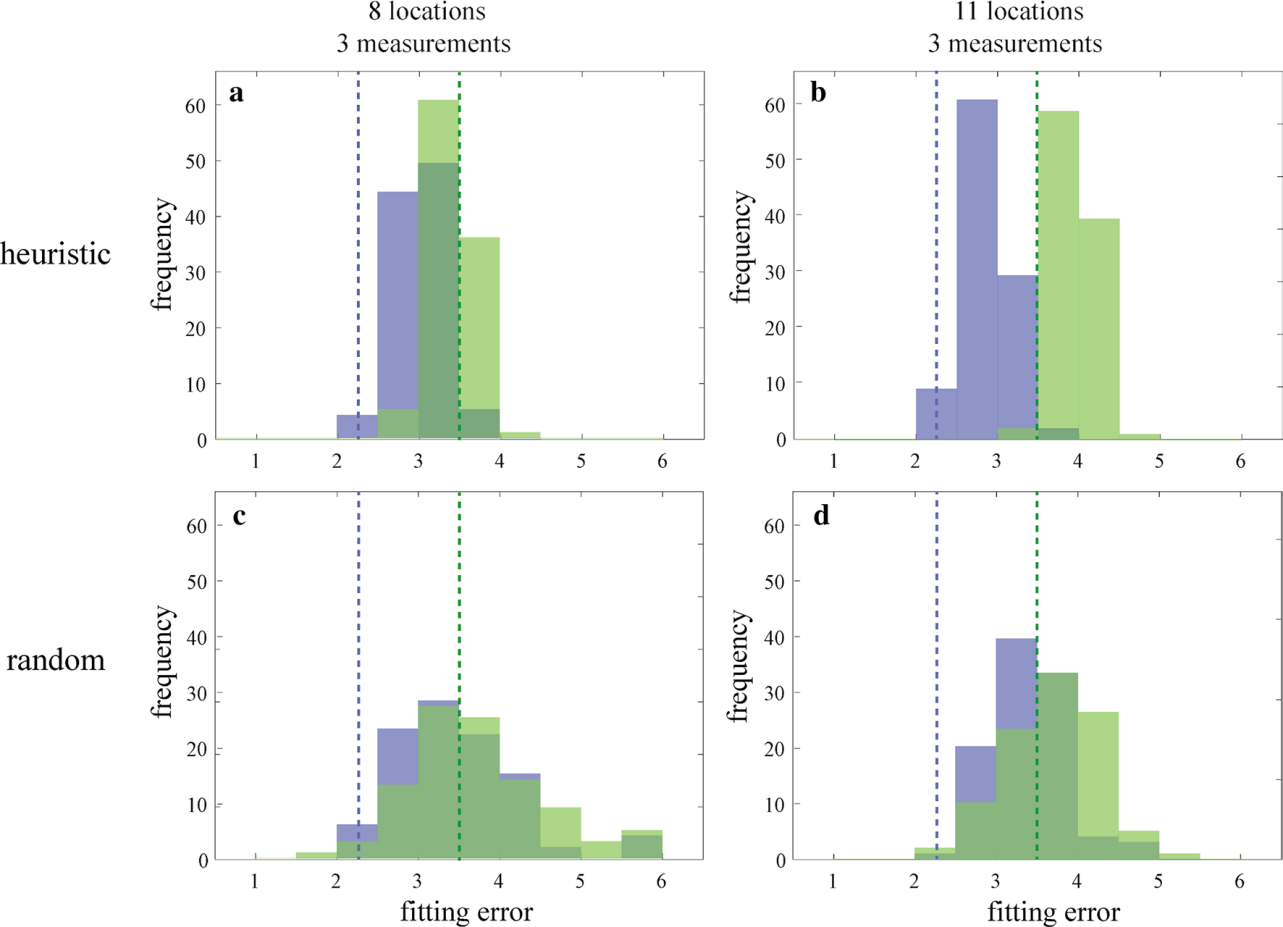
Generated distributions of final fitting error using heuristic (top row) versus random (bottom row) sampling strategies. Each distribution is the result of 100 iterations, with (**a**) 3 measurements at 8 evenly spaced locations; (**b**) 3 measurements at 11 evenly spaced locations; (**c**) 3 measurements at 8 randomly selected locations; (**d**) 3 measurements at 11 randomly selected locations. Iterations were run on datasets corresponding to the given hypothesis (blue) and the alternative-unknown hypothesis (green), and for each of the resulting generated datasets the final hypothesis fitting error was computed. The blue and green dashed vertical lines show the representative fitting error for the null and alternative dataset respectively, i.e., the fitting error if all possible (220) measurements in that dataset were collected

The generated final fitting error distributions for the heuristic versus random sampling strategies are shown in Fig. 6. Heuristics strategies in the present study offered a clear advantage in capturing data that were broadly representative of the overall statistical pattern of fit. On Fig. 6, this is evidenced by better convergence of the final fitting error to representative fit (marked by the vertical colored lines) with a heuristic strategy (A) relative to a random strategy (B)—it is particularly evident when locations are sampled at higher density (11 versus 8). Representative fit for each dataset is defined by the fitting error value if all possible (220) measurements were collected.

**Fig. 7:**
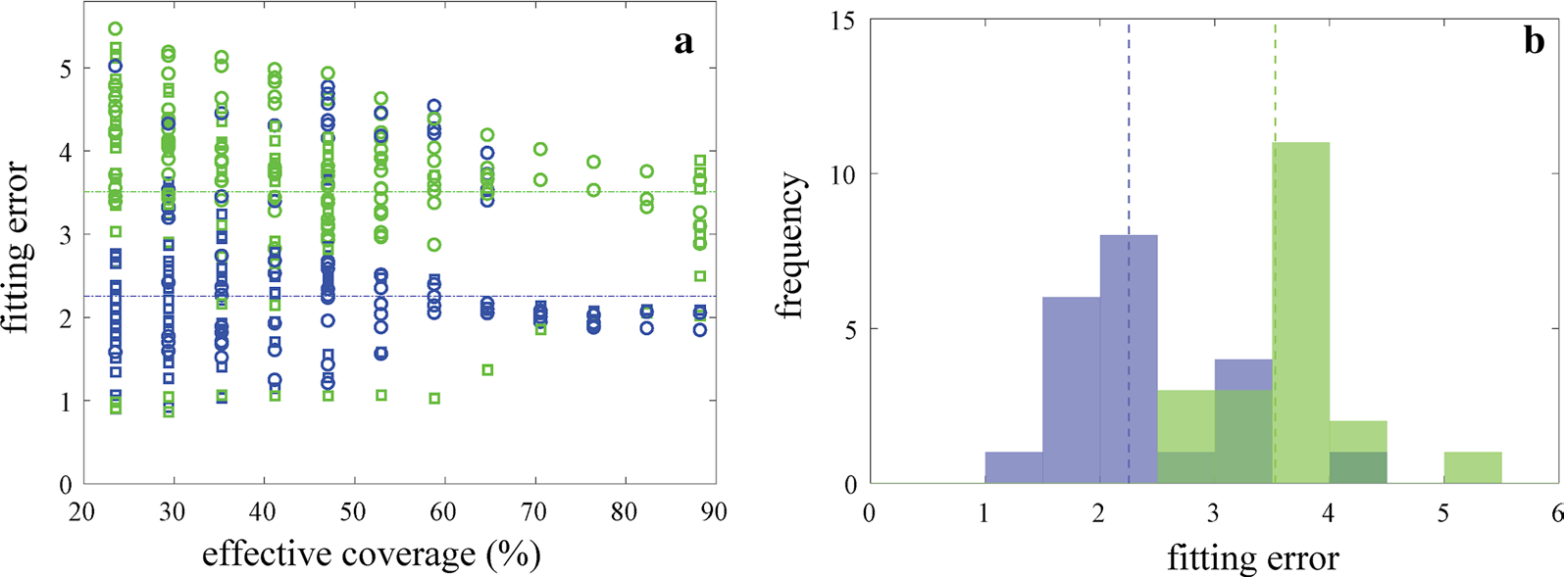
Expert geoscientists’ (**a**) fitting error by effective coverage, and (**b**) distribution of final fitting error. Fitting error was computed using the data each participant actually received, drawn from datasets corresponding to the given hypothesis (blue) and the alternative-unknown hypothesis (green). The dashed lines of the corresponding colors show the representative fitting error for each dataset, i.e., the fitting error if all possible (220) measurements in that dataset were collected

We also looked at the trajectory of hypothesis fitting error for each expert geoscientist participant based on whether they drew from a dataset that supported the given hypothesis or the alternative-unknown hypothesis. Fitting error was computed at each sampling step using the data the participant actually received. Figure 7a shows the trajectory of experts fitting error by percent effective coverage of the variable space, i.e., the ratio of intervals sampled in moisture content range.^8^ Experts’ strategies provide data that begins to converge on the representative fitting error (marked by the horizontal colored lines) around 60% effective coverage. This demonstrates that the sampling strategies expert geoscientists relied on allowed for efficient collection of high value data—a representative fitting error was reached by sampling just over half of the available variable-space.

Figure 7b shows the distributions of expert geoscientists’ final fitting error, based on whether they drew from a dataset that supported the given hypothesis or the alternative-unknown hypothesis. Again, we see good convergence of each expert distribution towards the representative fitting error (marked by the vertical colored lines). Note that geoscientists were not provided information on statistical convergence, but still had an intuitive understanding of convergence from the graphical data. This suggests that experts had a sense of when they had collected sufficient data to evaluate the given hypothesis; i.e., most geoscientists stopped collecting data when their fitting error reached a value that was representative.

Finally, we examined the degree to which expert geoscientists adapted their initial data collection strategies in response to incoming data. We were particularly interested in whether geoscientists would be susceptible to anchoring bias and rely too heavily on their initial strategies when given the opportunity to adjust data collection. Consistent with this, we found approximately 23% of geoscientists (9 of 39) did not adjust their initial strategy *at all*. Of these, 6 were assigned to receive data supporting the given hypothesis and 3 received data supporting the alternative-unknown hypothesis. Even among those who did adjust their strategies, there was still evidence of anchoring to initial location selections and the magic number. Approximately 77% of those who adjusted (23 of 30) waited until their initial strategy was complete before collecting additional data–12 from the given hypothesis condition and 11 from the alternative-unknown hypothesis condition. The other 23% deviated from their initial strategy before it was complete, showing behavior consistent with encounter-conditional search heuristics or area-restricted search (Hills et al., 2013; Pacheco-Cobos et al., 2019). But of those who deviated part-way through, the majority (5 of 7) ended up visiting all the locations in their initial strategy eventually. Approximately 67% of those who adjusted their initial strategy (20 of 30) kept using the same magic number–9 from the given hypothesis condition and 11 from the alternative-unknown hypothesis condition. Among those who did change their magic number, the overall frequency of changes was proportionally small—of all locations sampled, across all experts, only 16% (32 of 206) involved a change in magic number.

The fact that nearly all expert geoscientist participants showed some form of anchoring to initial heuristic strategies is neither surprising nor unreasonable given the general success of their heuristics at capturing high value data (as shown in Fig. 6). With continued reliance on heuristic strategies, the majority of experts (31 of 39) made the correct conclusion about the hypothesis for the dataset they sampled from, and all experts reported being either very confident or moderately confident in their conclusion. Although the number of experts who made an incorrect conclusion about the hypothesis is small, there are recurring similarities amongst their decision strategy that are worth brief discussion.

**Fig. 8:**
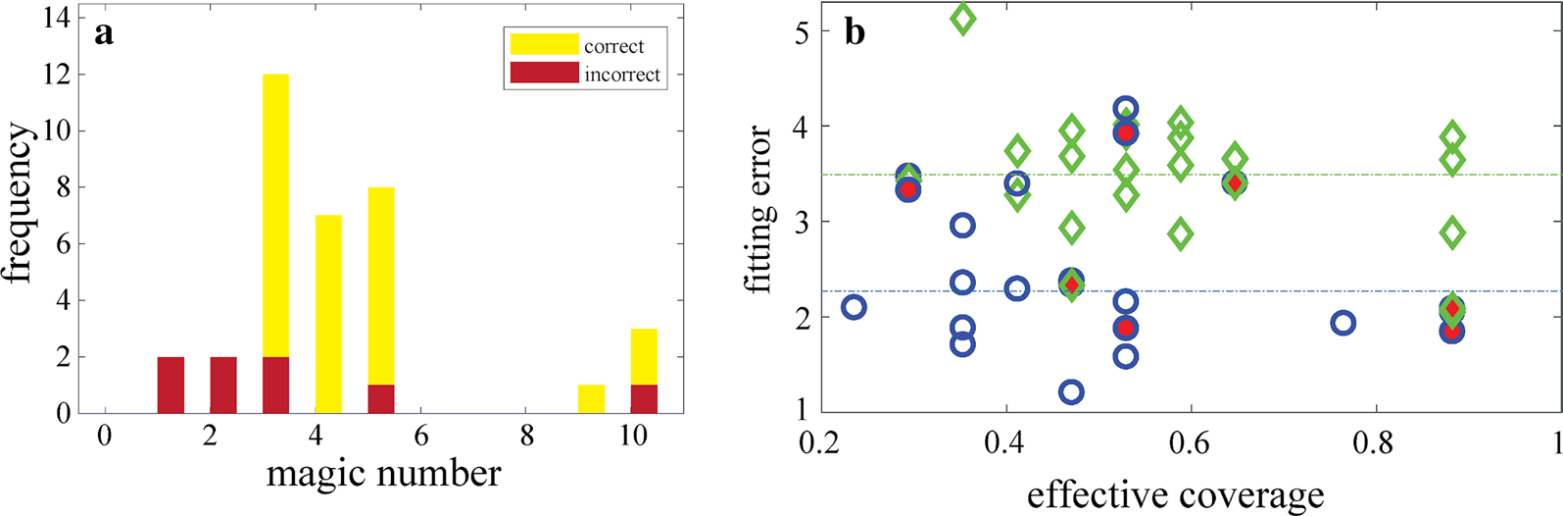
Characterization of expert sampling strategies that were associated with a Type I error. **a** Frequency of expert magic numbers plotted by experts who made correct (yellow) and incorrect (red) conclusions. **b** Expert fitting error computed at the conclusion step. Blue circles represent individuals who received data supporting the given hypothesis and green diamonds represent individuals who received data supporting the alternative-unknown hypothesis. Filled markers represent individuals who made the wrong conclusion. The dashed lines of the corresponding colors show the representative fitting error for each dataset

Amongst those who made the incorrect conclusion, all but one made a Type 1 error; i.e., experts sampled from the dataset that supported the given hypothesis, but made the conclusion to reject that hypothesis. Half (four of eight) relied on magic numbers of two or fewer (see Fig. 8a), which increased the likelihood of under-sampling and obtaining a statistical pattern of fitting error that was not representative of the source dataset. Also, half (four of eight) of the participants who made the wrong conclusion had insufficient coverage of the variable space (< 60%; see Fig. 8b), again reducing the probability of fitting error converging to the representative value. Taken together, these findings highlight the importance, for field scientists, of considering the variability at each site (i.e., the statistical distribution of noise) and the nature of spatial gradients (i.e., variable-space coverage).

In summary, the findings from our simulated geologic decision scenario suggest expert scientists are likely to use simple heuristics when making decisions about both where to collect data, and how much to collect—and the same heuristics are used, to a lesser degree, by novice undergraduates making data collection decisions. For the hypothesis and datasets used in the current experiment (based on real-world sediment dynamics), the heuristics experts relied on were efficient and effective at capturing high value data. Consequently, experts anchoring to their initial heuristics and only making small adaptations to their research strategy were not problematic. We discuss the scenario findings further in Sect. 5, including implications for scientific training and the use of mobile robotic platforms in field research.

## Discussion and conclusions

The findings from the case study and the simulated geologic decision scenario highlight the robust impact that heuristics can have on spatiotemporal data collection decisions. The equal spacing and magic number heuristics were generally successful, in both simulated and real-world field scenarios, at yielding data that allowed expert scientists to efficiently evaluate the hypothesis. This is good news for field scientists who rely on these heuristics – and we know many do, since there are recommended quality control practices for environmental and geological sampling that specify sampling at uniform intervals (U.S. Environmental Protection Agency, 2002), and taking an equal number of measurements at each location (Geboy & Engle, 2011; U.S. Geological Survey, 1987). Past work has shown that expert geologists sampling heuristics can be unsuccessful, however, failing to produce data that are representative of actual field conditions due to under-sampling (Gonzalez & Pasternack, 2015). Failure in data foraging has consequences, though blessedly not as severe as the life-or-death consequences associated with traditional foraging. The inadequate or incorrect application of data collection heuristics feeds forward into natural science interpretations, at best slowing scientific progress (e.g., the re-litigation of field methods and associated scientific conclusions based on larger datasets), and at worse impeding progress entirely (e.g., pre-paradigm shift intransigence; Kuhn, 2012).

Notably, the heuristics were successful in the current study because the environmental variables being assessed were normally distributed and had a gradual spatial gradient. One could imagine that if variables had a power law distribution (where a heavy tail makes large measurements more likely), or if a spatial gradient was highly non-linear, then the heuristics would be much less effective. Perhaps the fact that experts relied heavily on the heuristics reveals their underlying expectations about the typical nature of noise and spatial gradients in the natural world, i.e., noise is typically normally distributed and gradients are typically linear or gradual. Or it might be evidence of cultural transmission of foraging strategy, consistent with previous naturalistic data (McElreath & Koster, 2014) and laboratory research (Maya et al., 2019). Since novice undergraduates relied on the same heuristics (though to a lesser degree), this would suggest some combination of both, i.e., that expectations of normality and gradual spatial trends in the natural world are fundamental, but reinforced via cultural transmission in disciplinary geoscience.^9^ If so, this has strong implications for field science training—namely, that field scientists should be taught (a) to select data collection strategies based on the known properties of the variables of interest, and (b) to recognize the possible danger in using data collection strategies that are a poor fit for the environment. Training with simulated field scenarios that have known statistical properties offers a variety of pedagogical opportunities, including training scientists using a wide range of data conditions unconstrained by the need to find field sites or data with the desired statistical properties. Therefore, we believe simulated field scenarios, like the one used in the present study, have strong potential as tools for training scientists (novice and expert) about data collection decision making.

In both the case study and simulated scenario, geoscience experts demonstrated only limited adaptation of heuristic strategies in response to in-situ data. This form of “anchoring” to heuristics would be problematic in environments where the heuristics are a poor fit. However, as detailed in the preceding paragraph, the heuristics were broadly successful at assessing the variables of interest in the current study. Future research should examine the conditions under which expert scientists adapt data collection heuristics during dynamic hypothesis testing. One possibility is that heuristics only characterize the preliminary *exploratory* phase of hypothesis testing (where field scientists are seeking some minimum level of data required to test a plausible working hypothesis), but in the subsequent *verification* phase (where the aim of data collection is to improve confidence in the hypothesis to a desired level), heuristics are adapted or abandoned.

This distinction between exploratory and verification phases (Kartik et al., 2018) can be represented as separate inner-loop iterations of a dynamic hypothesis testing sequence. For example, in the case study, the expert geoscientist relied on heuristics during initial data collection (with little adaptation) to test the hypothesis, i.e., exploration phase—but once the general hypothesis was confirmed, the geoscientist indicated he would adapt their sampling strategy (abandoning the equal spacing heuristic) to provide a more precise test of the hypothesis, i.e., verification phase. In the simulated scenario, expert geoscientists were instructed to engage only in the exploration phase; however, when asked what future steps they would take to improve confidence in their conclusion, the majority responded that they would collect data along additional dune transects.^10^ Whether experts would continue relying on the same heuristics at new transects, or adapt their sampling strategy based on in-situ data (as occurred in the case study), is a question ripe for further study. Answering this question will not only require expansion of the environment (e.g., from single to multi-transect), but also a well-defined cost function for data collection. In the current study, the cost-function was subjective; participants were simply asked to make data collection decisions similar to how they would in the real-world (taking into account typical field constraints like time, opportunity loss, etc.). Moving forward, it is important that the cost function be objective to better evaluate different cognitive theories of search and foraging.

The current research focused on data collection decision making when scientists had a single working hypothesis. Yet, in geology, and other field sciences, scientists may approach a field area with multiple parallel or competing hypotheses, or without any specific well-defined hypothesis. In the latter case, field scientists instead use general strategies to guide data collection (e.g., equal space sampling; Reverdy et al., 2017) or make observations along paths that are parallel and perpendicular to regional geological gradients. It is an open question whether the heuristics observed in the current research will apply to such field conditions. Thus, an important area for future research on in-situ data collection decision making is contrasting data-driven and single-hypothesis versus multi-hypothesis-driven decisions.

The increasing use of technologies that offer in-situ data for field research, e.g., mobile robotic platforms, have the potential to transform and advance data collection practices beyond existing expert capabilities (Gil et al., 2018; Kimball et al., 2020; Shipley & Tikoff, 2019). In-situ data not only provide scientists the opportunity to make real-time adjustments to data collection strategies, but technologies may also be leveraged as decision support systems that (a) package and present data to users in a manner that improves processing fluency, and (b) provide guidance in the form of behavioral nudges (Wilson et al., 2019). For example, AI endowed with an a-priori hypothesis and data collection strategy could provide users with real-time statistical information on hypothesis fit while collecting data, and suggest revisions to the collection strategy based on accumulated data (e.g., “variability at this location is significantly higher than previous locations, would you like to collect more data before proceeding?”). We believe this type of collaborative and coordinated decision making in human–agent teams represents a possible future of field data collection and reasoning with data (cf. Alvard & Carlson, 2020). As technology grows, the structure of science, and what is required of the human scientist, will undoubtedly change. Understanding the cognitive underpinnings of adaptive data collection decisions will help inform the design of new technologies or workflows that promote optimal data foraging, and ultimately improve field science by better supporting scientists’ most important tools: their minds.

## Supplementary Information

**Additional file 1.** Description of dataset generation for simulated scenario.

## Data Availability

The materials and datasets used during the current study are available in the OSF repository, https://osf.io/yhpxs/.

## Footnotes

1 Rate maximization optimizes the difference between the benefits and costs per unit time: i.e., given a choice between different sites, the decision maker should select the option with the most value (if everything else is equal), and, when the value of the sites are equal, they should select the option that requires the least number of resources to acquire it (taking time into account).

2 Random search models have been a predominant, though highly controversial, approach to describing foraging behavior in human and non-human animals (Benhamou & Collet, 2015; Viswanathan et al., 2011). For example, many organisms display Lévy motion (Humphries, Weimerskirch, Queiroz, Southall, & Sims, 2012), where the length of move “steps” (distance traveled between two points) are drawn from a power law distribution with a heavy tail, resulting in clusters of short walks interspersed by long trajectories. However, the fact that random walks describe real-world movement patterns acceptably well should not be taken as evidence that the underlying mechanistic processes are also always random (Boyer, Miramontes, & Larralde, 2009; Fronhofer, Hovestadt, & Poethke, 2013).

3 RHex was selected for the current task because, relative to other terrestrial robots our team has access to (e.g., Spirit platform, Vasilopoulos et al., 2020; Minitaur platform, Topping et al., 2019), RHex has the longest established history of achieving robust and enduring locomotion in challenging terrains, like our wooded hillslope field site (Ilhan et al., 2018). We controlled RHex remotely with a human operator, rather than using an autonomous planner (Ilhan et al., 2018; Vasilopoulos et al., 2020) that relies on sensors and algorithms to find traversable open space and avoid unexpected contact or collision. Because the goal of our field outings was to assess expert human data collection, we opted to use the simplest possible configuration of the robot to achieve our locomotion goals. See our recent work on advancing robot mobility in natural environments that uses collisions and disturbances as opportunities to improve robot locomotion in complex environments (Qian & Koditschek, 2020).

4 Field data from the Wissahickon Valley Park, including the case study data and subsequent data collected using different sampling strategies, are viewable online in Notebook format: https://nbviewer.jupyter.org/github/cristinagwilson/data-foraging-in-human-robot-teams/blob/master/Field%20Work/WissahickonSoil.ipynb

5 At the time of participant recruitment, the new relationship between sand moisture and strength discovered by Qian et al. (2019) was not yet published. Still, to account for differences in initial knowledge we asked participants at the end of the task if they had any familiarity with the hypotheses or field setting. Although some participants were familiar with these features, they showed no consistent difference in performance from those who were unfamiliar.

6 The term “magic number” arose from discussions in the field with an expert geoscientist who, when observed taking the same number of measurements at each data collection site, was asked, “Is there a magic number of samples?”, to which the expert replied, “Yes! It’s four.”.

7 When we presented these results to a group of robotic engineers, they were shocked that more experts did not take the maximum data available, given there was no real cost to doing so in the simulated scenario. We believe this shows that experts were treating the scenario as akin to real field data collection, where there would be a cost associated with each data point (e.g., time, opportunity loss, sample analysis fee, etc.).

8 Variable-space coverage describes the volume of variable space filled out by the accumulated data relative to the possible range of variable values. Effective coverage was determined by splitting the range of possible variable values (for moisture) into 16 intervals and computing the ratio of intervals sampled.

9 An alternative possibility we considered is that expectations of normality and gradual/linear spatial trends in the natural world are a product of statistical training. However, amongst novice undergraduates, we found no difference in adherence to heuristics between those who had taken a statistics course and those who had not. Also, statistics curriculum is rare in geoscience programs (Manduca et al., 2008), making it unlikely that greater adherence to heuristics in experts versus novices is a product of greater statistical acumen.

10 It is standard practice for geoscientists to collect data at multiple environmental transects before drawing a conclusion about the variables under study. In field work at White Sands with the robot RHex and four dedicated researchers (Qian et al., 2019), it took an entire day (eight to ten hours) to sample a single transect from crest to interdunce (approximately 100 m), taking soil moisture and strength measurements at uniformly spaced intervals (every 10 m) with 4 measurements per location. In subsequent field outings in the Wissahickon with a smaller team and a better-refined workflow, we have been able to take more than twice the amount of data across a similarly sized space in almost half the time (five to six hours). Future simulated human–robot teaming data collection tasks should take into account the real-world constraints associated with using a mobile robot; however, one benefit of using such tasks is the flexibility to remove or lessen constraints, in anticipation of technological improvements that will allow for faster data collection.
