# Supplementary material for: Spatially and temporally distributed data foraging decisions in disciplinary field science
Source: Cogn Res Princ Implic. 2021 Apr 7;6:29. doi: 10.1186/s41235-021-00296-z (PMC8026803; doi:10.1186/s41235-021-00296-z)
Supplement: Supplementary file 1 — Additional file 1. Description of dataset generation for simulated scenario. [file 41235_2021_296_MOESM1_ESM.docx]

**Supplementary Material – Dataset Generation**

In the generated datasets, soil erodibility (*e*) and the spatial variation of moisture (*m*) are functions of the spatial location (*x*), defined as distance from the crest. For both hypotheses, we designed the average spatial variation of moisture to be small at locations near the crest ($0\leq x<a_{0}$) and then linearly increase along locations on the stoss slope towards the interdune area ($x>a_{0}$):

$$m_{0}(x)=\left\{ \begin{aligned} 0, 0\leq x<a_{0} \\ x-a_{0}, x>a_{0} \end{aligned} \right.$$

For the given hypothesis, we designed the spatial variation of soil erodibility ($e_{0}$) to be a small constant ($b_{1}$) at the dry crest locations ($0\leq x<a_{0}$). Soil erodibility increases linearly at locations where moisture begins to increase ($a_{0}\leq x<a_{1}$), before eventually becoming constant ($b_{2}$) at a location along the stoss slope where moisture reaches saturation ($x>a_{1}$):

$$e_{0}(x)= \left\{ \begin{aligned} b_{1}, 0\leq x<a_{0} \\ b_{1}+\frac{b_{2}-b_{1}}{a_{1}}*x, a_{0}\leq x<a_{1} \\ b_{2}, x>a_{1} \end{aligned} \right.$$

where $a_{0}$= 7, $b_{1}$= 2, $b_{2}$= 7.

For the alternative (unknown) hypothesis, we again designed soil erodibility ($e_{1}$) to be a small constant ($b_{3}$) at the dry crest locations ($0\leq x<a_{0}$). Soil erodibility still increases linearly at small moisture content locations on the stoss slope ($a_{0}\leq x<a_{2}$), i.e., where grains are slightly moist (Iveson et al., 2002). However, soil erodibility decreases at locations along the stoss slope ($a_{2}\leq x<a_{3}$), before becoming constant ($b_{5}$) at locations where moisture content approaches saturation ($x>a_{3})$.):

$$e_{1}(x)= \left\{ \begin{aligned} b_{3}, 0\leq x<a_{0} \\ b_{3}+\frac{b_{4}-b_{3}}{a_{2}}*x, a_{0}\leq x<a_{2} \\ \frac{b_{4}-b_{5}}{a_{2}-a_{3}}*x+b_{4}-\frac{b_{4}-b_{5}}{a_{2}-a_{3}}*a_{2}, a_{2}\leq x<a_{3} \\ b_{5}, x>a_{3} \end{aligned} \right.$$

where $a_{1}$= 14, $a_{2}$= 11, $a_{3}$= 13, $b_{3}$= 2, $b_{4}$= 12, $b_{5}$= 7.

To capture the variation in natural soil and instrument measurement noise, we generated the samples at each location with a truncated normal distribution around the average soil erodibility and moisture. For erodibility we used a standard deviation of $\pm$2 with truncating a bound of $\pm$1, whereas for moisture we used a standard deviation of $\pm$0.5 with a truncating bound of $\pm$0.5.
